# Four-gene signature predicting overall survival and immune infiltration in hepatocellular carcinoma by bioinformatics analysis with RT‒qPCR validation

**DOI:** 10.1186/s12885-022-09934-1

**Published:** 2022-07-30

**Authors:** Renguo Guan, Jingwen Zou, Jie Mei, Min Deng, Rongping Guo

**Affiliations:** 1 Department of Liver Surgery, Sun Yat-Sen University Cancer Center, 651 Dongfeng East Road, Guangzhou, China; 2 State Key Laboratory of Oncology in South China, Collaborative Innovation Center for Cancer Medicine, 651 Dongfeng East Road, Guangzhou, China

**Keywords:** Hepatocellular carcinoma, Signature, Nomogram, Prognosis, Immune infiltration

## Abstract

**Background:**

Hepatocellular carcinoma (HCC) is one of the most lethal cancers, with a poor prognosis. Prognostic biomarkers for HCC patients are urgently needed. We aimed to establish a nomogram prediction system that combines a gene signature to predict HCC prognosis.

**Methods:**

Differentially expressed genes (DEGs) were identified from publicly available Gene Expression Omnibus (GEO) datasets. The Cancer Genome Atlas (TCGA) cohort and International Cancer Genomics Consortium (ICGC) cohort were regarded as the training cohort and testing cohort, respectively. First, univariate and multivariate Cox analyses and least absolute shrinkage and selection operator (LASSO) regression Cox analysis were performed to construct a predictive risk score signature. Furthermore, a nomogram system containing a risk score and other prognostic factors was developed. In addition, a correlation analysis of risk group and immune infiltration was performed. Finally, we validated the expression levels using real-time PCR.

**Results:**

Ninety-five overlapping DEGs were identified from four GEO datasets, and we constructed a four-gene-based risk score predictive model (risk score = EZH2 * 0.075 + FLVCR1 * 0.086 + PTTG1 * 0.015 + TRIP13 * 0.020). Moreover, this signature was an independent prognostic factor. Next, the nomogram system containing risk score, sex and TNM stage indicated better predictive performance than independent prognostic factors alone. Moreover, this signature was significantly associated with immune cells, such as regulatory T cells, resting NK cells and M2 macrophages. Finally, RT‒PCR confirmed that the mRNA expressions of four genes were upregulated in most HCC cell lines.

**Conclusion:**

We developed and validated a nomogram system containing the four-gene risk score, sex, and TNM stage to predict prognosis.

**Supplementary Information:**

The online version contains supplementary material available at 10.1186/s12885-022-09934-1.

## Introduction

Hepatocellular carcinoma (HCC) is one of the most malignant digestive tumors, accounting for the third most frequent cancer mortalities in the world [1]. The Chinese people are deeply affected by HCC because there are large numbers of persons with hidden hepatitis B virus, and it is estimated that in 2018, there were 392.9 thousand newly diagnosed cases and 369 thousand HCC-associated deaths in China [2]. Among the treatments for HCC, surgery is still the predominant method. Moreover, most patients are already at an advanced stage when they are diagnosed with HCC. With the development of targeted therapy and immunotherapy, a large number of HCC patients can improve their conditions and fortunately have the opportunity to undergo surgical procedures. However, the overall 5-year survival of HCC patients is currently less than 20% [3]. Thus, there is an urgent need to identify prognostic biomarkers for HCC patients and further help clinical doctors make the best medical decisions.

The American Joint Committee on Cancer TNM staging system is widely applied in clinical practice [4]. Genetic alterations are widespread and contribute to the pathogenesis of HCC. However, due to the different molecular mechanisms of HCC, the prognosis of patients with the same stage may be different. In recent years, due to the rapid development of biotechnology, transcriptomics have promoted the identification of genetic candidates with prognostic value for HCC patients. In particular, some researchers constructed prognostic systems based on gene signatures. For example, Luo et al. constructed a prognostic model for HCC patients based on a 10-immune gene signature [5]. Xia et al. developed a powerful classifier to predict early-relapse based on a 24-mRNA signature [6]. Shi et al. established a stemness-based eleven-gene signature to predict the clinical outcomes of HCC patients [7]. However, these previous studies that only focused on a gene signature are still insufficient, and comprehensive analysis is required to further show its value.

We aimed to establish a novel and robust prediction signature that not only involves a minimum number of genes but is also combined with clinicopathological characteristics. In the present study, differentially expressed genes (DEGs) were identified from four publicly available Gene Expression Omnibus (GEO) datasets. Then, a four-gene signature and nomogram predicting overall survival were developed by The Cancer Genome Atlas (TCGA), and we examined the robustness of our results in the International Cancer Genomics Consortium (ICGC) cohort. Considering the importance of immune infiltration in the tumor microenvironment, we further investigated the differences in immune infiltration between high-risk and low-risk groups of HCC patients.

## Materials and methods

### Data acquisition and identification of DEGs

We searched the expression profiling by using arrays of HCC datasets in the GEO database, and the criteria for inclusion were as follows: Homo sapiens; arrays with more than 10 pairs of samples comparing the transcriptome profiles between tumor tissues and matched adjacent normal tissues. After carefully scrutinizing the GEO database (https://www.ncbi.nlm.nih.gov/geo/), we included four eligible datasets, namely, GSE45436 [8], GSE121248 [9], GSE101685, and GSE112790 [10]. First, the raw counts of the RNA-sequencing data were log2 transformed and quantile normalized. Next, we used the K-nearest neighbor method to fill in missing gene expression values. Then, the “Limma” R package was used to identify significant DEGs between the HCC and matched normal samples. The adjusted *P* values were calculated by using the Benjamini‒Hochberg false discovery rate method. An adjusted *P* value < 0.05 and |log2-fold change (FC)|≥ 2 were used as cutoff values for DEG inclusion. Ultimately, all DEGs from the four GEO datasets were merged to identify the upregulated and downregulated DEGs via the Venn tool.

### Functional enrichment analysis and construction of protein‒protein interaction (PPI) networks of DEGs

First, we selected the Database for Annotation, Visualization and Integrated Discovery (DAVID) (Version 6.8, https://david.ncifcrf.gov/) for functional enrichment analysis because it applies a comprehensive set of functional annotations for molecular functions (MFs), cellular components (CCs), biological processes (BPs) and Kyoto Encyclopedia of Genes and Genomes (KEGG) [11]. Functional enrichment with *P* values less than 0.05 was considered significant, and only the top six GO enrichment pathways and KEGG pathways were visualized using a chord plot. Next, protein‒protein interaction (PPI) networks of DEGs were constructed based on the Search Tool for the Retrieval of Interacting Genes (STRING) database (https://string-db.org/) [12]. Interactions with combined scores > 0.4 were considered statistically significant interactions. Next, the significant interactions were exported to Cytoscape software (version 3.8.2) for visual presentation and calculation of the top 10 hub genes.

### Screening for survival-associated DEGs and development and validation of a gene predictive signature

The overlapping DEGs obtained from the above analysis were further used to build a predictive gene signature. First, the RNA-seq data profiles of HCC and their corresponding clinical information were downloaded from the TCGA database (https://portal.gdc.cancer.gov/) and ICGC database (https://dcc.icgc.org/projects). The details of the four GEO datasets are listed in Table 1. Data from TCGA database were regarded as the training group. Next, univariate Cox regression analysis and multivariate Cox regression analysis based on the Akaike information criterion (AIC) were performed to identify survival-associated DEGs with the “survival” R package (*P* < 0.05). Then, based on the survival-associated DEGs obtained from the above analysis, we utilized the “glmnet” R package to conduct least absolute shrinkage and selection operator (LASSO) regression Cox analysis (simulation times = 1,000), and a risk score model based on gene mRNA values and coefficients of four DEGs was constructed. Furthermore, we divided the TCGA cohort into a high-risk group and low-risk group based on the mean value of the risk scores. Kaplan‒Meier survival curves were utilized to confirm the association between the risk score model and overall survival (OS). Next, a time-dependent receiver operating characteristic curve (time-dependent ROC) was drawn to evaluate the predictive ability of this predictive model based on the “timeROC” R package. To confirm its role as an independent prognostic factor, we conducted univariate Cox regression analysis and multivariate Cox regression analysis. We also investigated the association between risk score group and clinicopathological characteristics. Finally, the risk score model constructed by the TCGA cohort was further validated in the ICGC cohort.

**Table 1:** Clinicopathological characteristics of HCC patients in the TCGA cohort and ICGC cohort

	Characteristic	Group	N
TCGA cohort	Age (years)	≤ 60	167
		> 60	171
	Gender	male	231
		female	107
	Fustat	alive	224
		deceased	114
	Grade	G1	45
		G2	166
		G3	115
		G4	12
	T stage	T1	170
		T2	84
		T3	74
		T4	10
	TNM Stage	stage I	168
		stage II	83
		stage III	83
		stage IV	4
ICGC cohort	Age (years)	≤ 68	120
		> 68	123
	Gender	male	182
		female	61
	Fustat	alive	199
		deceased	44
	TNM Stage	stage I	37
		stage II	109
		stage III	75
		stage IV	22

### Development and validation of a nomogram system

To develop a nomogram prediction system to predict the 1-year, 3-year, and 5-year OS probabilities of HCC patients in the TCGA cohort, three independent prognostic factors, including sex, TNM stage and risk score, were sequentially subjected to a stepwise Cox regression model. Next, we plotted a calibration curve to assess the consistency between the observed rates and predicted OS. At the same time, we drew time-dependent ROC curves to compare the predictive performance of different prognostic factors. Finally, this nomogram prediction system was further validated in the ICGC cohort.

### Gene set enrichment analysis (GSEA)

GSEA is often used for the analysis and interpretation of genome-wide expression profiles [13]. Two gene sets databases: h. All. V7.4 Symbols.gmt (Hallmarks) and c2.cp.kegg.V7.4 Symbols.gmt (Curated) were downloaded from MSigDB (http://www.gsea-msigdb.org/gsea/msigdb/index.jsp). Then, we used GSEA software to identify the enriched signaling pathways based on the DEGs between the high-risk and low-risk groups in the TCGA cohort. Significantly enriched pathways were defined as those with *P* values <0.05, |normalized enrichment scores (NES)|> 1, and false discovery rates (FDR) <0.25.

### Correlation analysis of risk group and immune infiltration in the tumor microenvironment

Considering the important role of immune infiltration in tumorigenesis and progression, we first used the “e1071” and “parallel” R packages to analyze the infiltration levels of 22 types of immune cells in HCC tissues. Gene set signatures for each immune cell type were downloaded from CIBERSORTx (https://cibersortx.stanford.edu/index.php) [14]. Then, we analyzed the difference in immune infiltration between the high-risk and low-risk groups based on the Wilcoxon test. Finally, we investigated the correlation analysis between risk scores and 22 types of immune cells based on Spearman’s correlation analysis.

### Cell culture

Human HCC cell lines (e.g., SNU-449, HCCLM3, Hep-3B, HepG2, SK-Hep-1, MHCC97-H, PLC-8024, HuH7) were cultured in Dulbecco’s modified Eagle’s medium (DMEM, Gibco Invitrogen, Carlsbad, CA, USA) supplemented with 10% fetal bovine serum (Gibco). All cell lines were purchased from the Cell Lines Service (Cellcook Biotech Co., Ltd., Guangzhou, China).

### RNA extraction, reverse transcription, and quantitative reverse transcription PCR

Total RNA was isolated using an RNA quick purification kit (ESscience, China) and reverse-transcribed to cDNA using reverse transcription performed using a Fast All-in-One RT Kit (ESscience, China). Super SYBR Green qPCR Master Mix (ESscience, China) was used for RT‒PCR according to the manufacturer’s instructions. The qPCR primers are listed in Supplementary Table 2. The relative expression levels of five necroptosis-related genes were compared with that of β-actin, and the 2^−△△ct^ method was utilized to calculate the fold changes.

### Statistical analysis

R software (version 4.1.0), GraphPad software (version 8) and SPSS software (version 20) were used for statistical analysis. The associations between the risk score groups and clinicopathological characteristics were assessed using the χ^2^ test or Fisher's exact test. *P* values < 0.05 were considered significant in the present study.

## Results

### Identification of DEGs between HCC and adjacent normal tissue

**Fig. 1:**
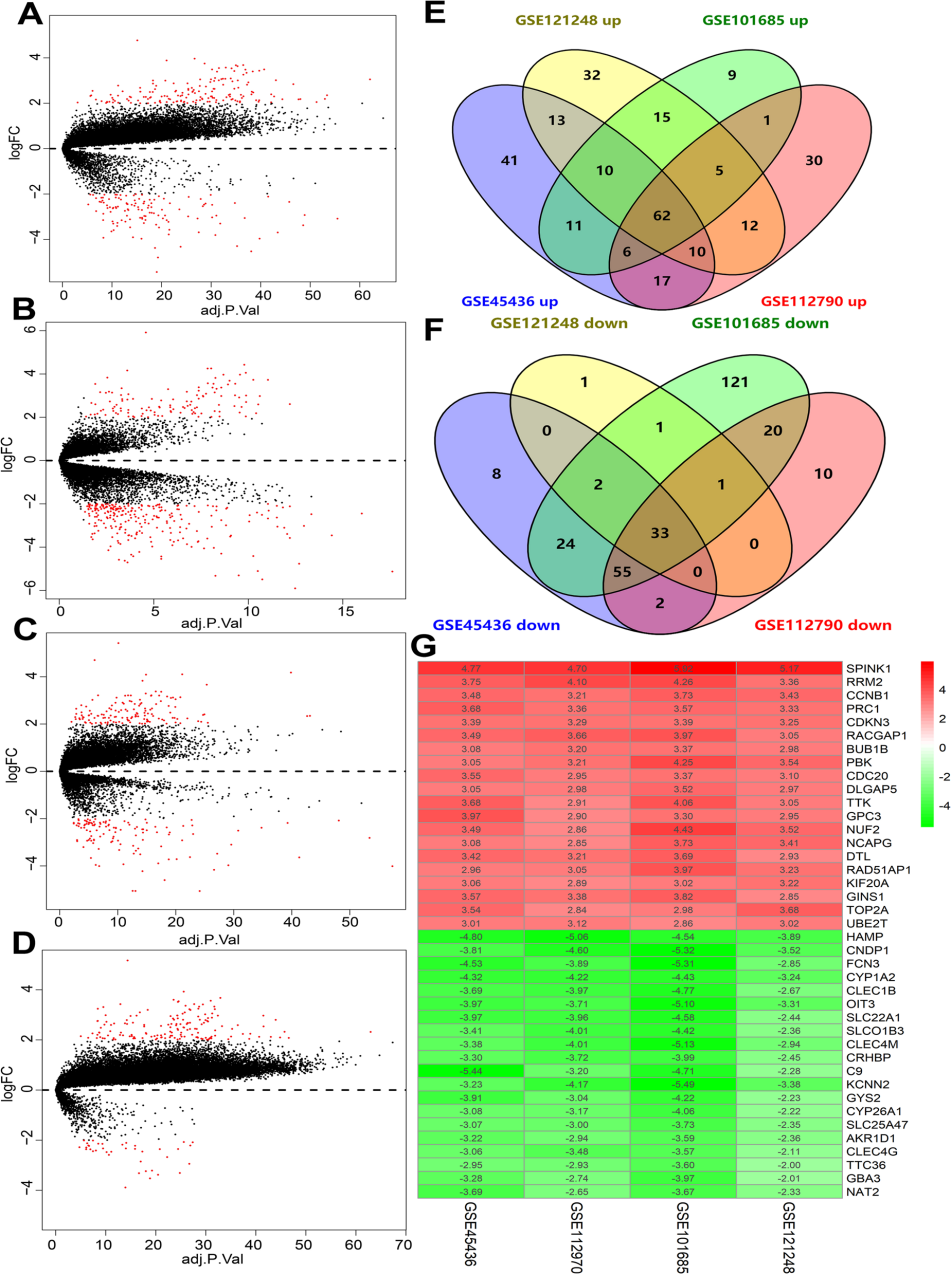
Identification of differentially expressed genes (DEGs) in GEO datasets. (**A**) volcano plots from GSE45436, (**B**) volcano plots from GSE101685, (**C**) volcano plots from GSE112790, (**D**) volcano plots from GSE121248, (**E**) venn plots from upregulated DEGs from four included GEO datasets, (**F**) venn plots from downregulated DEGs from four included GEO datasets, and (**G**) heatmap of the top 20 downregulated and upregulated DEGs. Green and red colors represent the down- and upregulated |FC| values, respectively. Values of *P* < 0.05 and lg|FC|≥ 2 were considered significant

Four GEO datasets were analyzed by the HG-U133_Plus_2 platform. Based on the cutoff values mentioned above, the DEGs in these four GEO datasets were identified (Fig. 1A-D). The overlapping DEGs were merged via a Venn diagram, and as shown in Fig. 1E, F, there were 62 upregulated DEGs and 33 downregulated DEGs. The list of overlapping differentially expressed genes (DEGs) is shown in Supplementary Table 1. The top 20 downregulated and upregulated DEGs are presented in Fig. 1G.

### Functional enrichment and signal pathway analysis

**Fig. 2:**
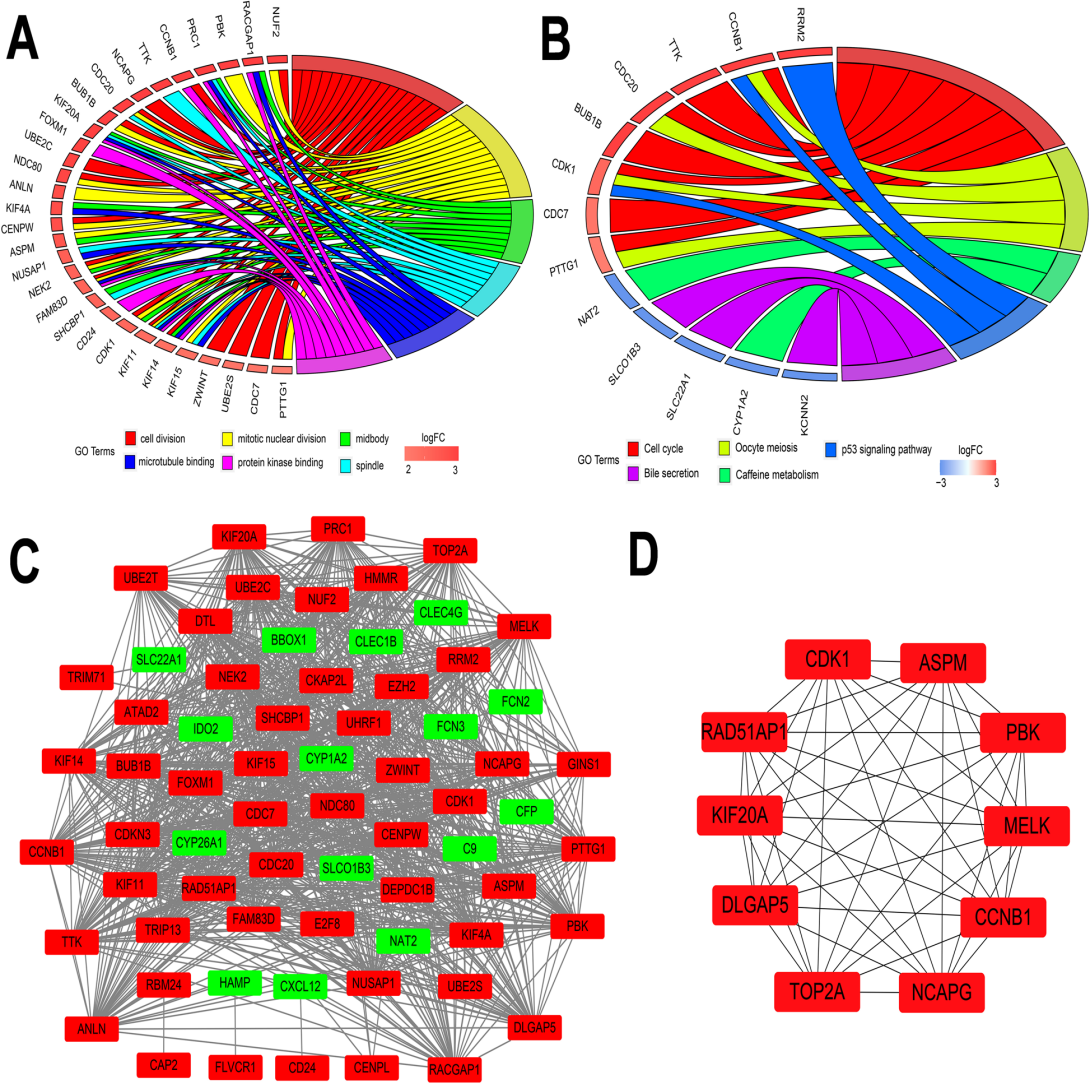
The top six significant enrichment GO terms and KEGG pathways of reference genes for all DEGs in HCC and protein‒protein interaction (PPI) networks of DEGs. Chord plot depicting the reference genes for all DEGs associations via ribbons to their assigned GO terms (**A**) and KEGG pathways (**B**). Colored rectangles represent the logFC values of genes. PPI networks(**C**) and hub genes (**D**) were visualized by cytoscape software. Green and red colors represent the downregulated DEGs and upregulated DEGs, respectively

In our study, the DAVID database was used for functional enrichment analysis of the overlapping DEGs. The gene ontology results suggested cell division and mitotic nuclear division in BP, midbody and spindle in CC, and microtubule binding and protein kinase binding in MF (Fig. 2A). The KEGG pathway analysis indicated that cell cycle, oocyte meiosis, caffeine metabolism, p53 signaling pathway, and bile secretion were significantly enriched (Fig. 2B). Collectively, we concluded that these overlapping DEGs might be involved in the development of HCC.

### Construction of PPI networks of DEGs and hub genes analysis

To explore the functions of these genes, we used the STRING database to construct PPI networks of these overlapping DEGs. The results indicated that the PPI networks contained 68 nodes and 937 edges (Fig. 2C). Hub gene analysis was further conducted by the cytoHubba application in Cytoscape, and maternal embryonic leucine zipper kinase (MELK), PDZ binding kinase (PBK), DNA topoisomerase II alpha (TOP2A), cyclin-dependent kinase 1 (CDK1), abnormal spindle microtubule assembly (ASPM), kinesin family member 20A (KIF20A), RAD51 associated protein 1 (RAD51AP1), DLG associated protein 5 (DLGAP5), non-SMC condensin I complex subunit G (NCAPG), and cyclin B1 (CCNB1) were regarded as the top hub genes (Fig. 2D).

### Development and validation of a four-gene risk score predictive signature

**Fig. 3:**
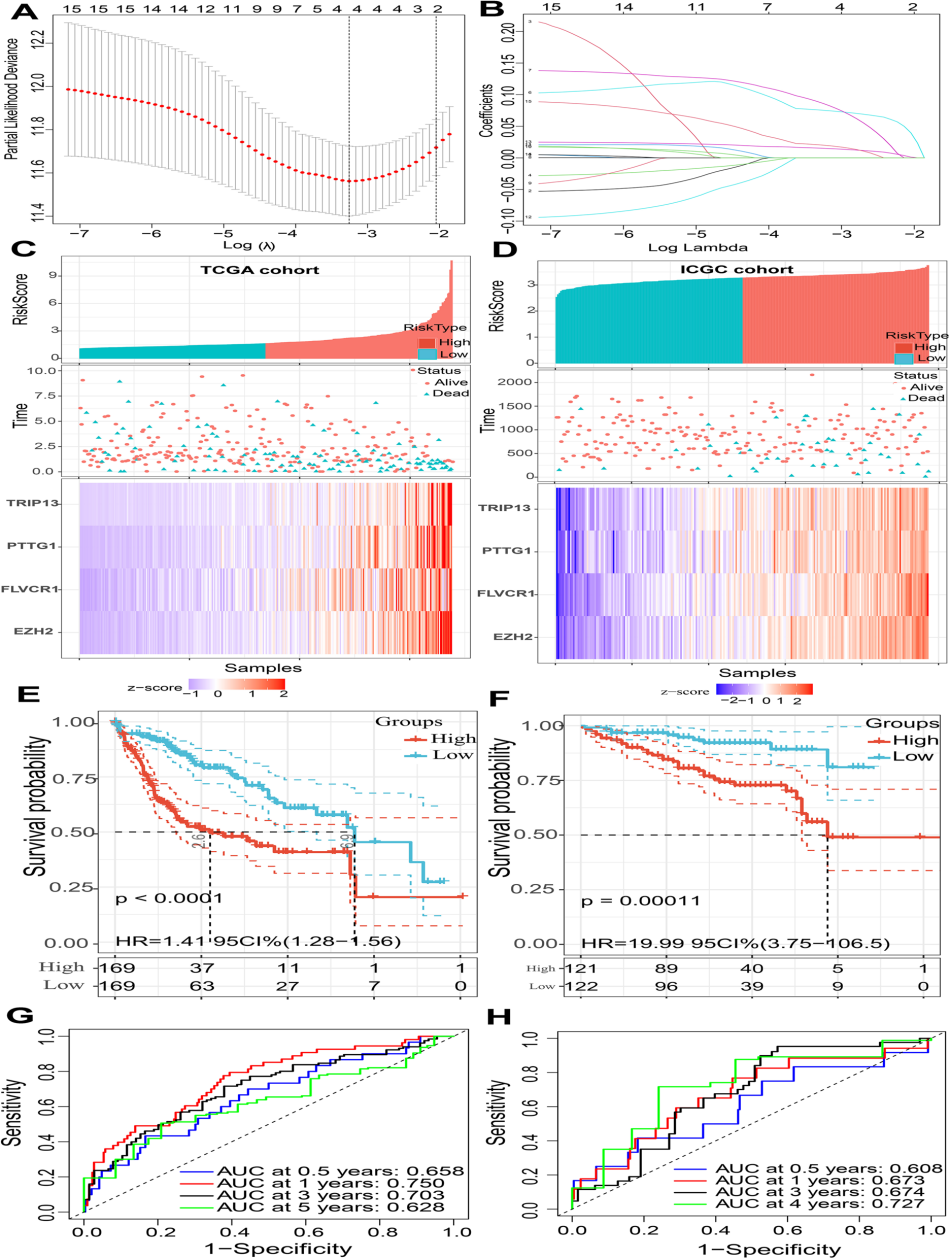
Construction of a prognostic model of four DEGs in the training cohort (TCGA cohort) and validation of that in the validation cohort (ICGC cohort). Cross-validation to find the optimal lambda value in the LASSO regression (**A**). LASSO regression analysis was performed to select radiomic features for prognostic model-building for HCC patients. Feature coefficients were plotted against the shrinkage parameter (Lambda) (**B**). Risk score analysis of the four-gene-based signature. Risk score distributions (top), survival overviews (middle), and heatmaps (bottom) for patients assigned to high-and low-risk groups based on the risk scores in the TCGA cohort (**C**) and ICGC cohort (**D**). Kaplan‒Meier estimates of the overall survival (OS) using the prognostic model for patients in the TCGA cohort (**E**) and ICGC cohort (**F**) Time-dependent receiver operating characteristic (ROC) curves for the survival of high- and low-risk groups in the TCGA cohort (**G**) and ICGC cohort (**H**). Different colors represent different years

To establish a risk score predictive signature, univariate Cox regression analysis and multivariate Cox regression analysis based on the Akaike information criterion (AIC) were performed to identify survival-associated DEGs in the TCGA cohort. A total of 16 independent survival-associated DEGs were identified (Supplementary Table 2). Then, these independent survival-associated DEGs were subjected to LASSO regression Cox analysis, and a 4-gene signature that can predict OS in HCC patients was developed: enhancer of zeste 2 polycomb repressive complex 2 (EZH2), feline leukemia virus subgroup C cellular receptor 1 (FLVCR1), pituitary tumor-transforming 1 (PTTG1), and thyroid hormone receptor interactor 13 (TRIP13) (Fig. 3A-B). The results suggested that all 4 genes had positive coefficients. The prognostic risk score for each patient in the TCGA cohort was calculated by using a combination of gene expression level values and coefficients of all four DEGs (risk score = EZH2 * 0.075 + FLVCR1 * 0.086 + PTTG1 * 0.015 + TRIP13 * 0.020). Furthermore, the TCGA cohort and ICGC cohort were divided into a high-risk group and low-risk group, respectively, according to the mean value of the risk scores. As shown in Fig. 3C-F, Kaplan‒Meier curve analysis revealed that there were significantly different survival times between the high-risk group and low-risk group in the TCGA cohort and ICGC cohort, and compared with the low-risk scores, high-risk scores indicated a poor prognosis (P < 0.05). Finally, a time-dependent ROC plot was drawn to evaluate the predictive ability of this four-gene predictive model. The area under the curve (AUC) is associated with model performance. Notably, the AUCs of this four-gene predictive model for 0.5, 1, 3 and 5 years were 0.658, 0.750, 0.703, and 0.628, respectively, in the TCGA cohort (Fig. 3G). The AUCs of this four-gene predictive model for 0.5, 1, 3 and 4 years were 0.608, 0.673, 0.674, and 0.727, respectively, in the ICGC cohort (Fig. 3H). The AUC values indicated that our risk score prediction model has good sensitivity and specificity.

### Role as an independent prognostic factor and correlation with clinicopathological characteristics

**Fig. 4:**
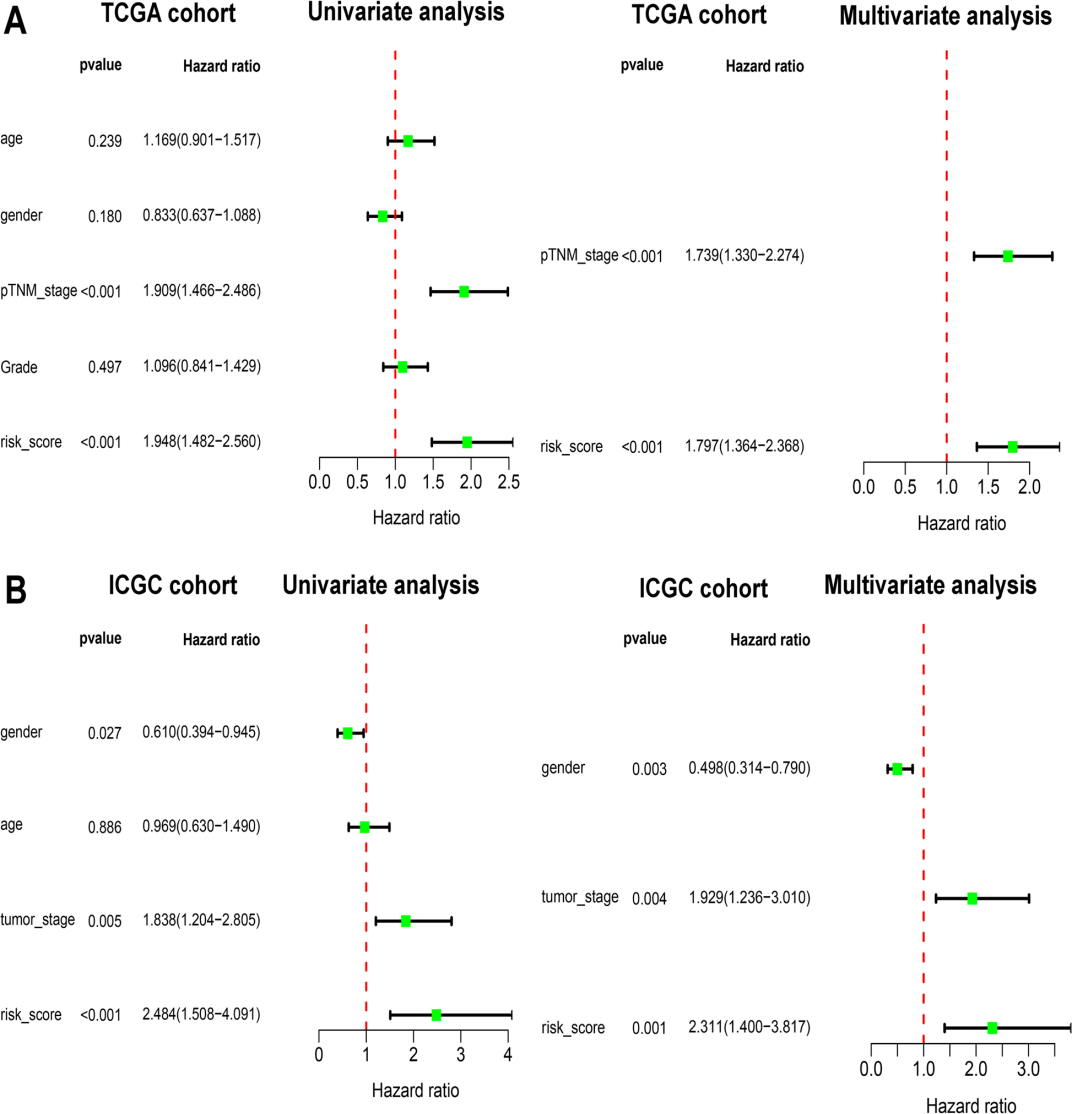
Risk score is an independent prognostic factor for HCC patients. Univariate cox regression analysis and multivariate cox regression analysis were conducted. Forest plot of univariate cox regression and multivariate cox regression of the risk scores in the TCGA cohort (**A**) and ICGC cohort (**B**)

**Fig. 5:**
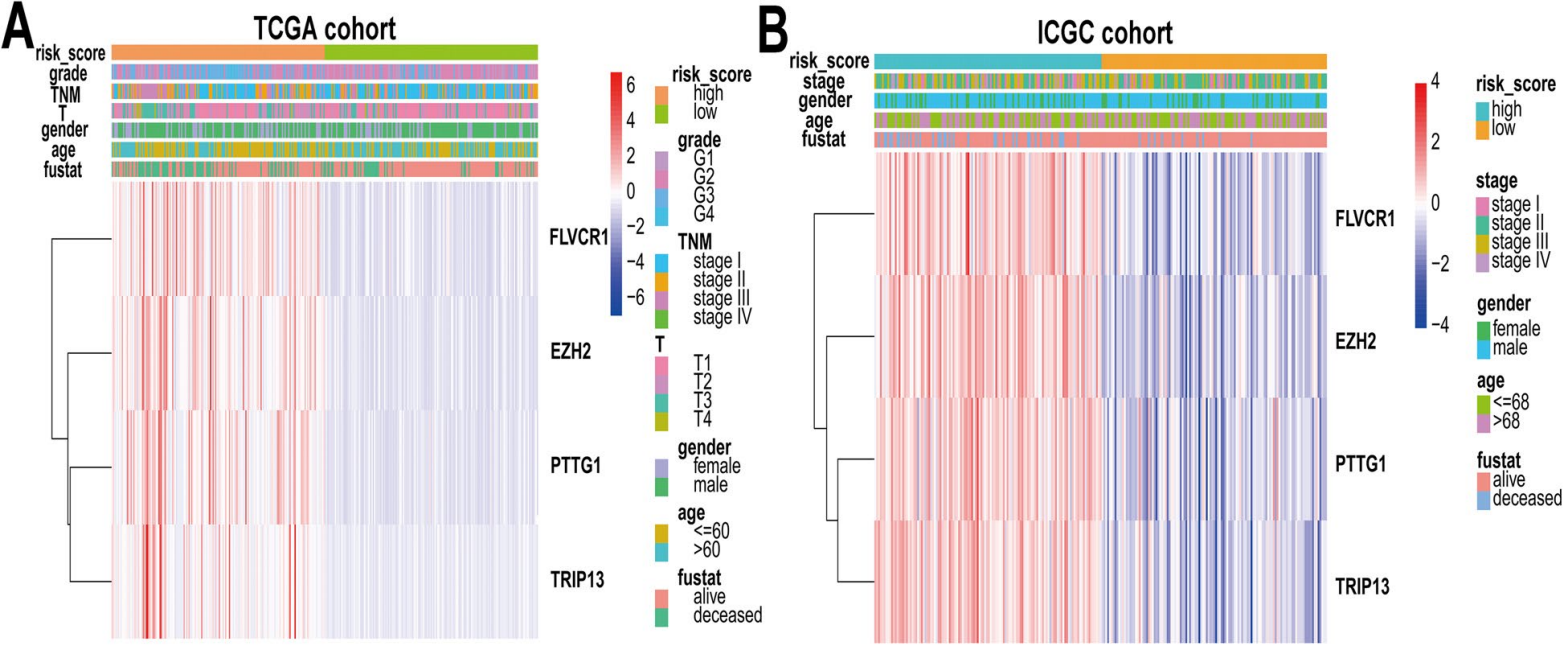
Heatmap of signatures and clinical parameters of HCC patients in the TCGA cohort (**A**) and ICGC cohort (**B**)

High-risk scores were associated with poor OS, and we conducted univariate Cox regression analysis and multivariate Cox regression analysis to further confirm their role as an independent prognostic factor. As shown in Fig. 4A, B, four-gene risk scores and TNM stages were considered independent prognostic factors in the TCGA cohort, and sex, TNM stage and four-gene risk scores were considered independent prognostic factors in the ICGC cohort (*p* < 0.05). We also investigated the association between the risk score group and clinicopathological characteristics. The details of the clinicopathological characteristics of each patient are presented with a heatmap (Fig. 5A, B). Table 2 shows that the results suggested that risk scores were significantly associated with age, T stage, TNM stage, and grade in the TCGA cohort and that there was a significant association between risk scores and TNM stages in the ICGC cohort.

**Table 2:** Correlations between risk scores and clinicopathological characteristics in the TCGA cohort and ICGC cohort

	Parameters	Risk score		χ^2^	P
		Low	High		
TCGA cohort	Age			9.9541	0.0016^a^
	≤ 60	69	98		
	> 60	100	71		
	Gender			2.3110	0.1285^a^
	male	122	109		
	female	47	60		
	Grade			43.9071	< 0.0001^a^
	G1-G2	135	76		
	G3-G4	34	93		
	T stage			9.1249	0.0025^a^
	T1-T2	139	115		
	T3-T4	30	54		
	TNM Stage			8.1880	0.0042^a^
	I-II	137	114		
	III-IV	32	55		
ICGC cohort	Age			0.2024	0.6528^a^
	≤ 68	58	62		
	> 68	63	60		
	Gender			0.1654	0.6842^a^
	male	92	90		
	female	29	32		
	TNM Stage			5.9368	0.0148^a^
	I-II	82	64		
	III-IV	39	58		

^a^ Pearson chi-square test

^b^ Continuity modified chi-square test

^c^ Fisher's exact test

### Development and validation of a nomogram system

**Fig. 6:**
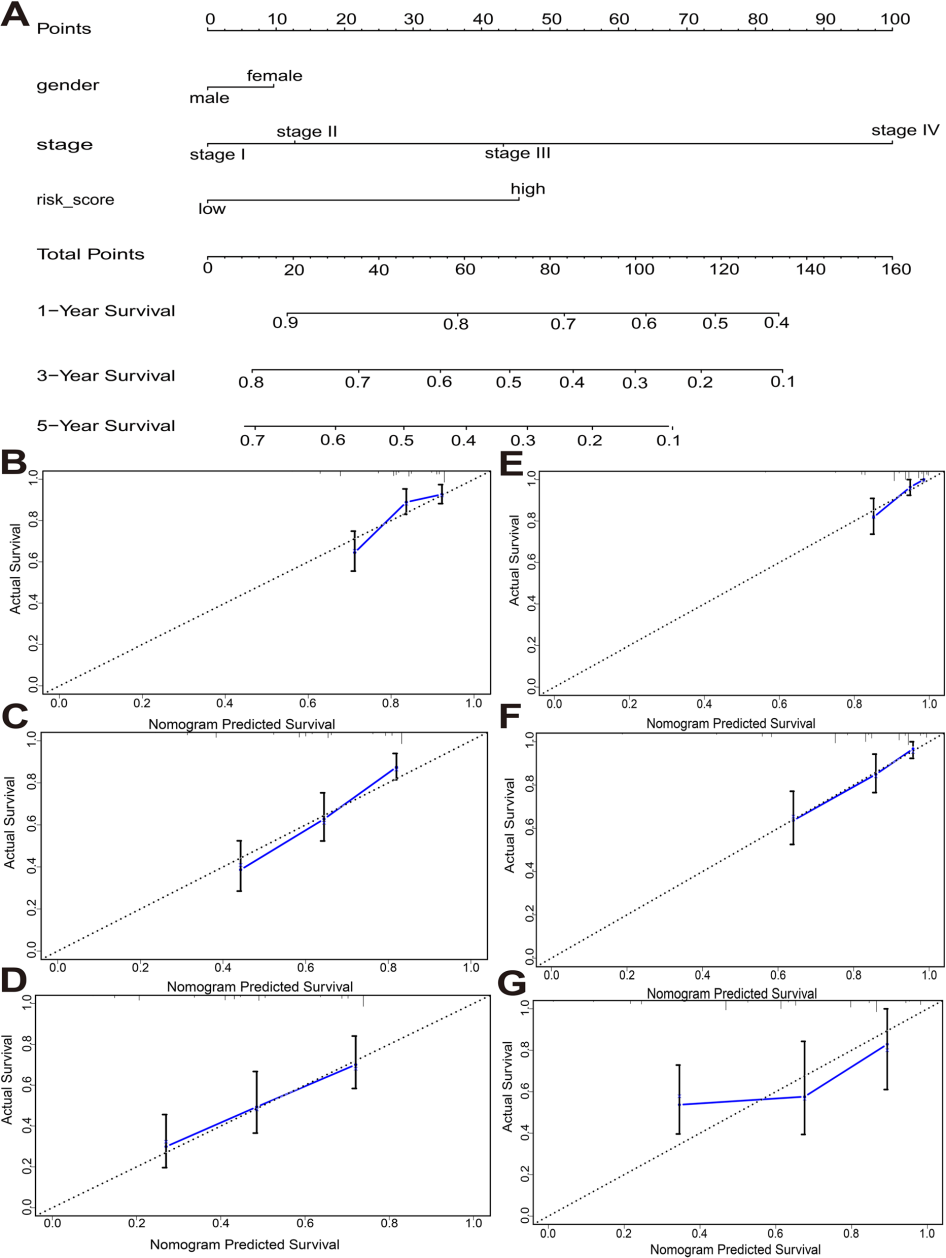
Construction of the nomogram predicting overall survival in the training cohort (TCGA cohort) and validation in the validation cohort (ICGC cohort). (**A**) nomogram predicting 1-, 3-, and 5-year overall survival of HCC. Calibration curve for predicting 1-, 3-, and 5-year overall survival of HCC in the TCGA cohort (**B-D**). Calibration curve for predicting 1-, 3-, and 4-year overall survival of HCC in the ICGC cohort (**E-G**). The y axis represents actual survival, and the x axis represents the predicted survival

**Fig. 7:**
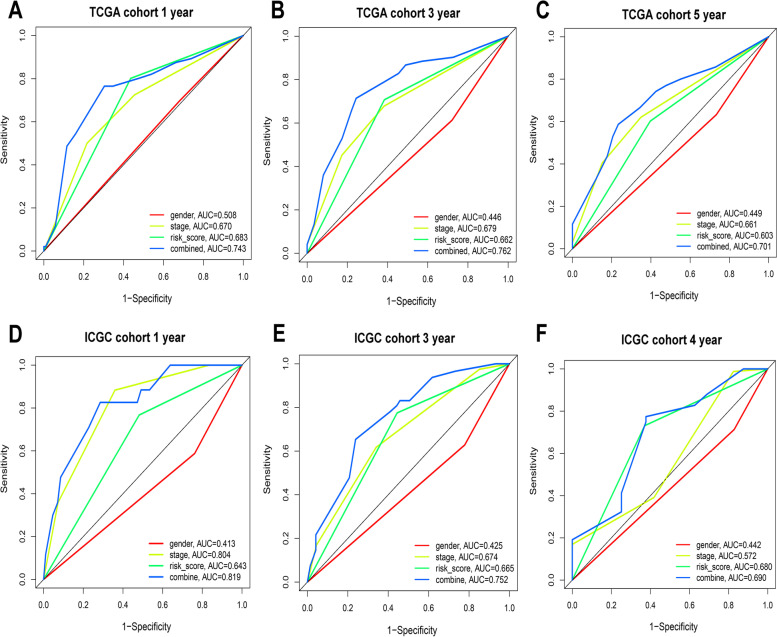
Time-dependent ROC curve for the nomogram. The prognostic accuracy of the nomogram system was further assessed by using comparisons to other prognostic clinical factors, and the AUC values for each model in the TCGA cohort (**A**) and ICGC cohort (**B**) are shown. Different colors represent different parameters and models

Three independent prognostic factors, including sex, TNM stage and risk score, were sequentially subjected to a stepwise Cox regression model to develop a nomogram prediction system in the TCGA cohort (Fig. 6A). Meanwhile, a nomogram prediction system was also validated by the ICGC cohort. The calibration curve for predicting the 1-year, 3-year, and 5-year OS probabilities of HCC patients in the TCGA cohort indicated that the nomogram-predicted survival closely corresponded with actual survival outcomes (Fig. 6B-D). The calibration curve of the ICGC cohort also indicated that the nomogram containing three independent prognostic factors performed well (Fig. 6E-G). Finally, we drew time-dependent ROC curves to compare the predictive performance of the nomogram system with other prognostic factors, including sex, TNM stage and risk score. Notably, as shown in Fig. 7A-F, the performance of the nomogram system was significantly better than that of sex, TNM stage and risk score.

### GSEA of biological functions between the high-risk and Low-risk groups

**Fig. 8:**
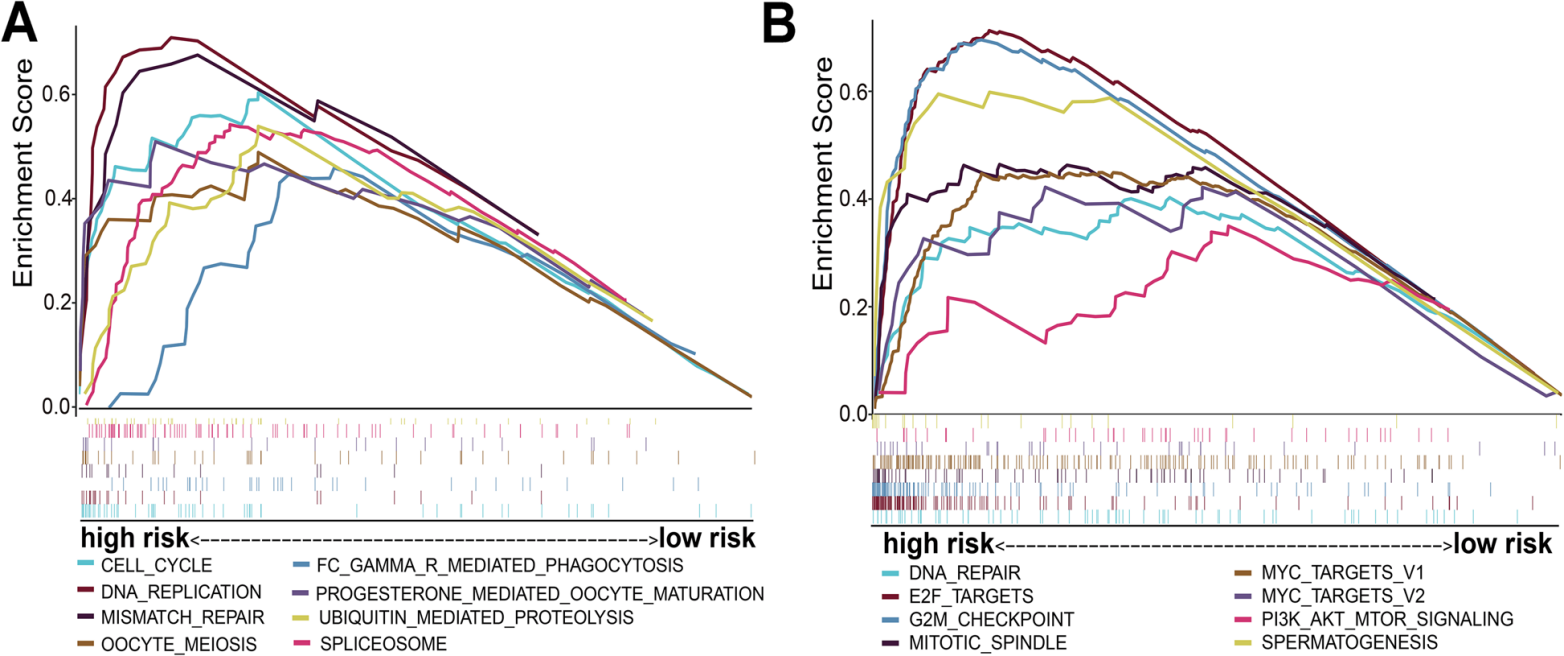
Gene set enrichment analyses (GSEA) of the different biological functions between the high-risk and low-risk groups. The top eight Kyoto encyclopedia of genes and genomes (KEGG) pathways in high-risk group via GSEA (**A**). The top eight hallmark enrichment pathways in the high-risk group via GSEA (**B**)

GSEA was used to further explore different biological functions between the high-risk and low-risk groups. Figure 8A shows that the top eight KEGG signaling pathways in the high-risk patients were cell cycle, DNA replication, Fc gamma R-mediated phagocytosis, mismatch repair, oocyte meiosis, progesterone-mediated oocyte, spliceosome, and ubiquitin-mediated proteolysis. Meanwhile, we found that the top eight hallmark enrichment pathways were DNA repair, E2F targets, G2/M checkpoint, mitotic spindle, MYC targets V1, MYC targets V2, PI3K-AKT-mTOR signaling and spermatogenesis (Fig. 8B).

### Correlation analysis of risk group and immune infiltration

**Fig. 9:**
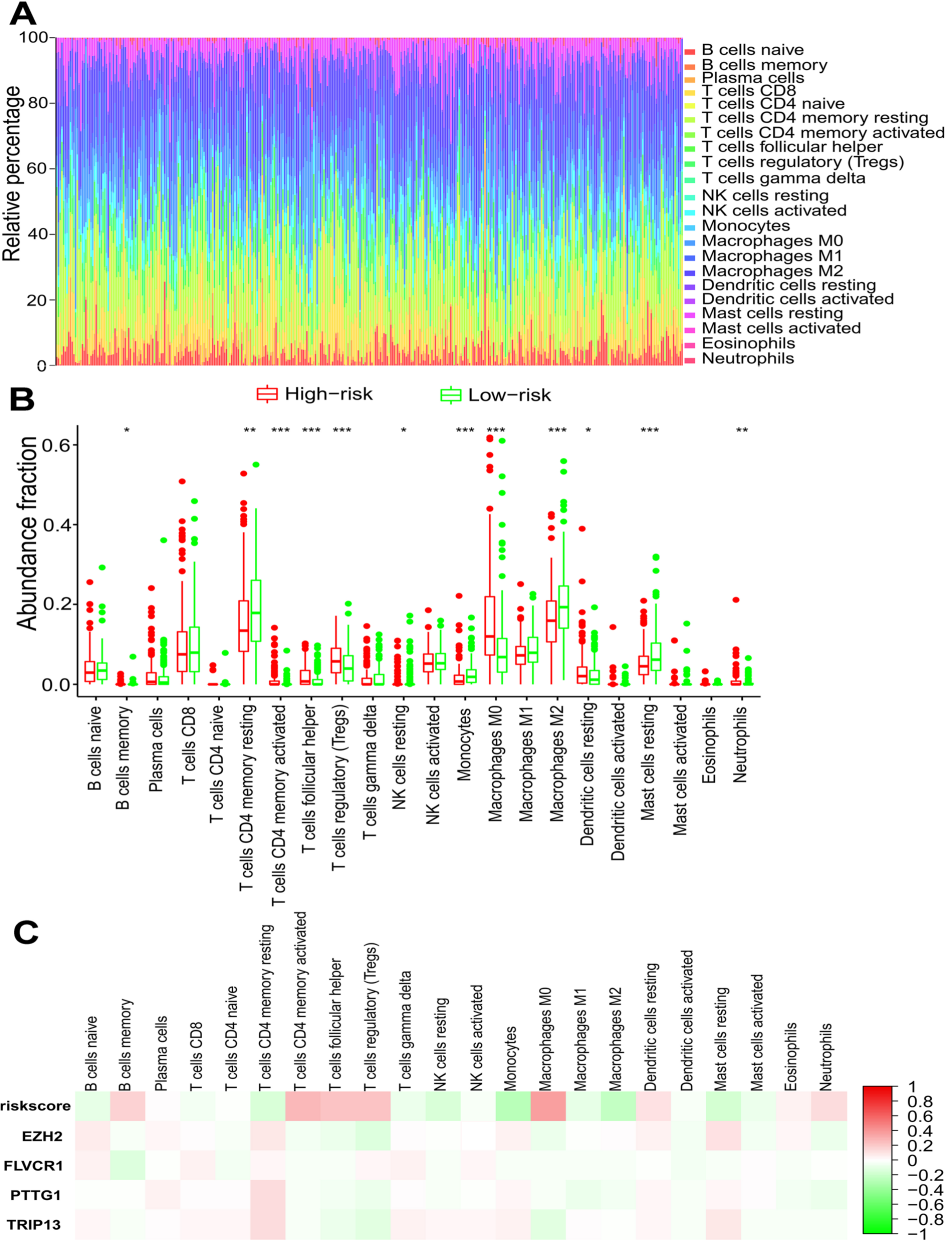
Correlation analysis of risk group and immune infiltration. The abundance fractions of 22 kinds of immune infiltration cells in the TCGA cohort (**A**). Each column represents one sample, and different colors represent different cells. The height of the color in each column represents the abundance score of immune infiltration cells in this sample. The box plot shows the different immune infiltration values between high-risk and low-risk group patients (**B**). Green and red colors represent low-risk and high-risk groups, respectively. *P < 0.05; **P < 0.01; and ***P < 0.001. A heatmap shows the correlation matrix of risk scores, four signature genes, and relative abundances of 22 kinds of immune infiltration cells (**C**). Green and red colors represent negative correlations and positive correlations, respectively

Considering that GSEA indicated that the high-risk group patients were associated with Fc gamma R-mediated phagocytosis and that immune infiltration plays a vital role in tumors, we further performed correlation analysis of risk groups and immune infiltration. Figure 9A shows the infiltration of 22 types of immune cells in HCC tissues. Moreover, there were significantly different B-cell memory, resting memory CD4 T cells, activated memory CD4 T cells, follicular helper T cells, regulatory T cells (Tregs), resting NK cells, monocytes, M0 macrophages, M2 macrophages, resting dendritic cells, resting mast cells and neutrophil infiltration properties between the high-risk and low-risk groups (Fig. 9B). Finally, a correlation analysis between risk scores and 22 types of immune cells also confirmed these differences (Fig. 9C).

### RT‒qPCR validation of four genes in HCC cell lines

**Fig. 10:**
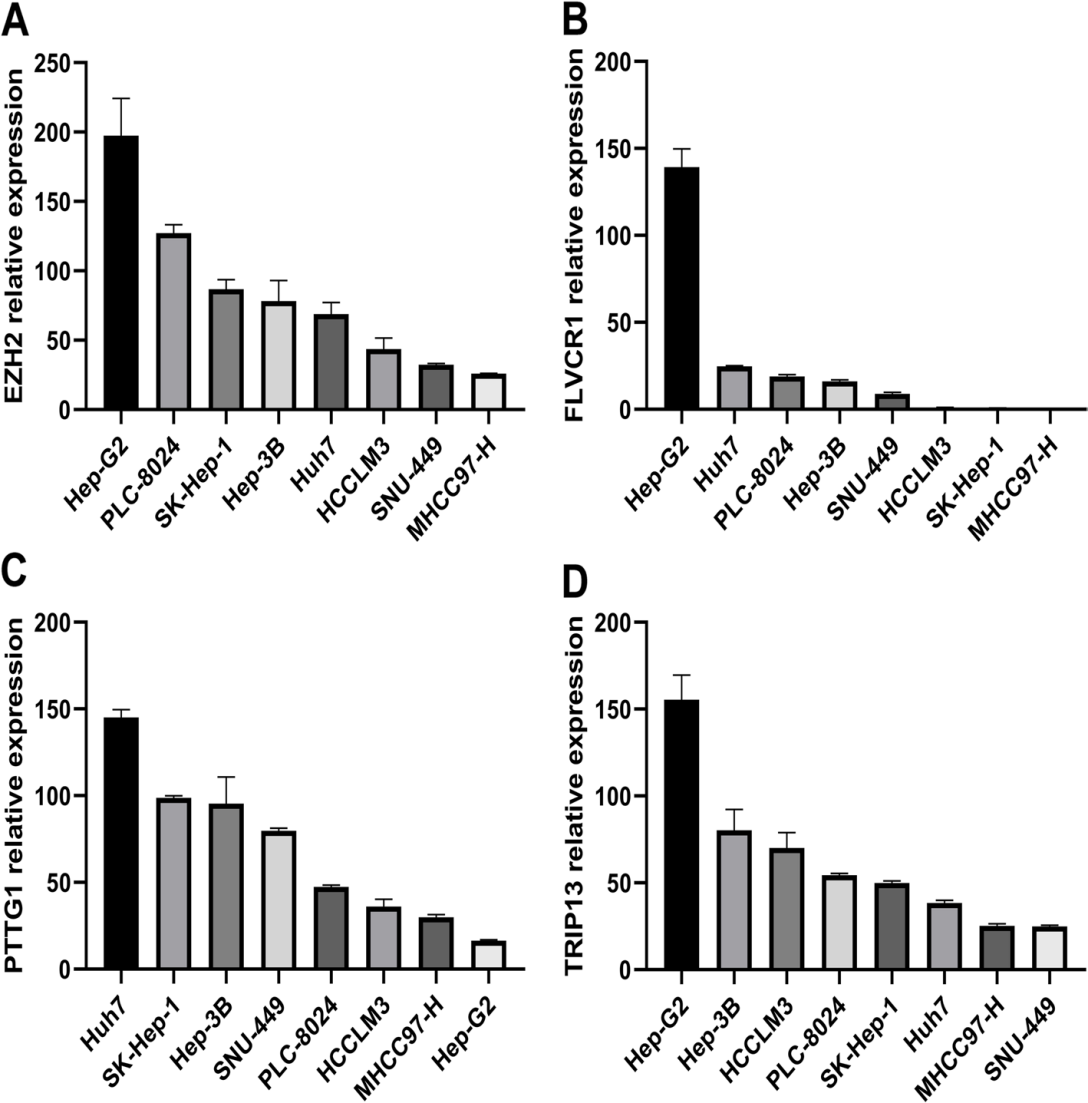
The mRNA expression levels of four genes in HCC cell lines. EZH2 (**A**), FLVCR1 (**B**), PTTG1 (C) and TRIP13 (**D**)

We used RT‒qPCR to further verify the expressions of four genes in the HCC cell lines. The results showed that the expressions of four genes were upregulated in most HCC cell lines. The relative expressions of EZH2, FLVCR1 and TRIP13 in HepG2 cells were obviously higher than those in the others, and PTTG1 had the highest expression levels in Huh7 cells (Fig. 10A-D).

## Discussion

In the present study, we established a four-gene risk score signature and constructed a nomogram containing risk score, sex and TNM stage to improve the ability to predict HCC prognosis. Moreover, we found that the four-gene risk score signatures were associated with immune cell infiltration.

Based on 95 overlapping DEGs obtained from four GEO data databases, we constructed a four-gene risk score model. Previous studies have confirmed that EZH2 contributes to cisplatin resistance and sorafenib resistance [15, 16]. Moreover, EZH2 was associated with the malignant transformation of oral leukoplakia, and the latest research suggested that EZH2 could be targeted by the long noncoding RNA, RC3H2, which could be a complementary endogenous RNA sponging miR-101-3p that further facilitates cell proliferation and invasion in oral squamous cell carcinoma [17, 18]. FLVCR1 regulates the development of synovial sarcoma by inhibiting apoptosis and autophagy, but its role in HCC still needs to be determined [19]. Elevated upregulation of PTTG1 activated by lncRNA PTTG3P promotes tumor growth and metastasis [20]. Upregulated TRIP13, interacting with ACTN4, induces progression of HCC by driving the AKT/mTOR pathway [21]. A study showed that TRIP13 is also related to the EMT pathway in lung cancer [22]. TRIP13 can promote tumor growth and metastasis in a p53-independent and MSI-independent manner [23]. Interestingly, GSEA of biological function between the high-risk and low-risk groups suggested that the high-risk group was mostly enriched with pathways involved in cell growth and the immune microenvironment. This further suggests the rationality of the risk score model. We found that risk scores were associated with CD4 + T cells, Treg cells, monocytes, NK cells and M2 macrophages. Notably, a previous study demonstrated that EZH2 is negatively related to immune infiltration. Inhibition of EZH2 can reduce the recruitment of regulatory T cells (Tregs), thereby reducing the activity of Tregs and enhancing T-cell infiltration in tumors to enhance antitumor immunity [24]. Inhibition of EZH2 leads to an increase in the transcription level of NKG2D, an NK-cell ligand, and increases NK-cell-mediated cytotoxicity against HCC cells [25]. Moreover, there is a relationship between EZH2 and immune checkpoint inhibitors. EZH2 can negatively regulate PD-L1 expression by increasing the promoter H3K27me3 levels of CD274 and IRF1 in HCC cells [26]. EZH2 inhibits the immunogenicity and antigen presentation of melanoma cells. EZH2 inhibition can cooperate with anti-CTLA-4 and IL-2 immunotherapy to inhibit the growth of melanoma [27]. Collectively, risk score signatures might help clinical doctors to identify those patients who can benefit from immunotherapy. However, the potential relationships among the remaining four genes and the immune microenvironment are still unknown.

The nomogram system using the risk score, sex and TNM stage might have a better ability to predict prognosis than the TNM stage model. Compared with most previous studies, the present study had some differences. First, LASSO regression Cox analysis was used for the identification of a gene-based signature. By constructing a penalty function, it can compress the coefficients of genes and make some regression coefficients zero, thereby achieving the goal of screening for highly relevant genes [28]. Second, our risk score prediction model consisted of only four genes, while those in previous studies often consisted of many genes. Third, we also developed and validated a nomogram system using risk score, sex and TNM stage, which might have good predictive performance. Finally, immunotherapy plays an indispensable role in the treatment of advanced HCC, and there is an urgent need to identify biomarkers for predicting immunotherapy responses. Consistent with a previous study, we also conducted correlation analysis of risk groups and immune infiltration, which might provide a strategy for immunotherapy [29].

We must acknowledge potential limitations in our analysis. First, there was only one external validating cohort with a small number of HCC patients. Second, the potential mechanism between risk scores and immune microenvironments should be further investigated by in vitro and animal experiments.

## Conclusions

We developed and validated a nomogram system using a four-gene risk score, sex, and TNM stage to improve HCC management regarding the ability to predict prognosis. Moreover, the risk score signature might help clinical doctors identify those patients who can benefit from immunotherapy.

## Supplementary Information

**Additional file 1: Table 1.** Primers of four genes for RT‒Qpcr.

**Additional file 2: Table 2.** List of the overlapping differentially expressed genes (DEGs).

**Additional file 3: Table 3.** Univariate Cox regression analysis and multivariate Cox regression analysis based on Akaike Information Criterion (AIC) of differentially expressed genes (DEGs).

## Data Availability

The datasets generated and analyzed during the current study are available in the [Gene Expression Omnibus] repository, [https://www.ncbi.nlm.nih.gov/geo/], [The Cancer Genome Atlas] repository, [https://portal.gdc.cancer.gov/] and [International Cancer Genomics Consortium] repository, [https://dcc.icgc.org/].
