# Supplementary material for: Four-gene signature predicting overall survival and immune infiltration in hepatocellular carcinoma by bioinformatics analysis with RT‒qPCR validation
Source: BMC Cancer. 2022 Jul 30;22:830. doi: 10.1186/s12885-022-09934-1 (PMC9338612; doi:10.1186/s12885-022-09934-1)
Supplement: Supplementary file 1 — Additional file 1: Table 1. Primers of four genes for RT‒Qpcr. [file 12885_2022_9934_MOESM1_ESM.docx]

Supplementary Table 1. Primers of four genes for RT‒qPCR.

| Target | Sequence (5’–3’) |
| --- | --- |
| FLVCR1 (F) | GAAAAACCTCGGTATCCACCAA |
| FLVCR1 (R) | CTAGCGTTAGCCCAATCCTTC |
| PTTG1 (F) | TGATCCTTGACGAGGAGAGAG |
| PTTG1 (R) | GGTGGCAATTCAACATCCAGG |
| EZH2 (F) | AATCAGAGTACATGCGACTGAGA |
| EZH2 (R) | CTGTATCCTTCGCTGTTTCC |
| TRIP13 (F) | TGTGTAAAGCGTTAGCCCAGA |
| TRIP13 (R) | GCCACTTTCCGAAAACCACTTA |
| GAPDH (F) | CCCACTCCTCCACCTTTGAC |
| GAPDH (R) | CCCACTCCTCCACCTTTGAC |
