# Supplementary material for: Four-gene signature predicting overall survival and immune infiltration in hepatocellular carcinoma by bioinformatics analysis with RT‒qPCR validation
Source: BMC Cancer. 2022 Jul 30;22:830. doi: 10.1186/s12885-022-09934-1 (PMC9338612; doi:10.1186/s12885-022-09934-1)
Supplement: Supplementary file 2 — Additional file 2: Table 2. List of the overlapping differentially expressed genes (DEGs). [file 12885_2022_9934_MOESM2_ESM.docx]

Supplementary Table 2. List of the overlapping differentially expressed genes (DEGs).

| DEGs | Gene name |
| --- | --- |
| Upregulated genes | AKR1B10, TRIM71, PTTG1, KIF14, C12orf75,CAP2, ZWINT, SULT1C2, NEK2, KIF15, KIF11, CKAP2L, CDK1, CENPL, CD24, RBM24, ATAD2, CDC7, NUSAP1, TRIP13, SHCBP1, ANLN, FLVCR1, DEPDC1B, CRNDE, CTHRC1, E2F8, FAM83D, CENPW, ASPM, RAD51AP1, EZH2, COL15A1, CLGN, UBE2T, HMMR, PBK, DLGAP5, KIF20A, NCAPG, BUB1B, NDC80, KIF4A, UBE2S, UBE2C, CDKN3, FOXM1, DTL, MELK, CCNB1, NUF2, RACGAP1, TOP2A, CDC20, GINS1, UHRF1, TTK, PRC1, RRM2, PEG10, GPC3, SPINK1 |
| Downregulated genes | C9, HAMP, FCN3, MT1M, CYP1A2, LINC01093, SLC22A1, OIT3, GYS2, CNDP1, APOF, CLEC1B, NAT2, SLCO1B3, CLEC4M, CRHBP, GBA3, KCNN2, AKR1D1, RSPO3, CYP26A1, SLC25A47, CLEC4G, HAO2, TMEM27, IDO2, CFP, FLJ22763, CLRN3, NPY1R, CXCL12, FCN2, BBOX1 |
