# Supplementary material for: Four-gene signature predicting overall survival and immune infiltration in hepatocellular carcinoma by bioinformatics analysis with RT‒qPCR validation
Source: BMC Cancer. 2022 Jul 30;22:830. doi: 10.1186/s12885-022-09934-1 (PMC9338612; doi:10.1186/s12885-022-09934-1)
Supplement: Supplementary file 3 — Additional file 3: Table 3. Univariate Cox regression analysis and multivariate Cox regression analysis based on Akaike Information Criterion (AIC) of differentially expressed genes (DEGs). [file 12885_2022_9934_MOESM3_ESM.docx]

Supplementary Table 3. Univariate Cox regression analysis and multivariate Cox regression analysis based on Akaike Information Criterion (AIC) of differentially expressed genes (DEGs).

|  |  | Univariate Cox analysis | | | Multivariate Cox analysis | | | |
| --- | --- | --- | --- | --- | --- | --- | --- | --- |
| Gene | HR | Lower.95.CI | Upper.95.CI | *P*-value | HR | Lower.95.CI | Upper.95.CI | *P*-value |
| UBE2C | 1.143193657 | 1.095535587 | 1.192924953 | 7.29E-10 | 1.129381034 | 0.986287743 | 1.293234686 | 0.078371519 |
| RACGAP1 | 1.234492638 | 1.131315744 | 1.347079348 | 2.24E-06 | 1.239271418 | 1.095098619 | 1.402424969 | 0.00067487 |
| FLVCR1 | 1.119353486 | 1.057024631 | 1.185357643 | 0.000114719 | 0.693269018 | 0.563935402 | 0.852264159 | 0.000506305 |
| PBK | 1.000517504 | 1.00020356 | 1.000831546 | 0.001233053 | 1.000520044 | 1.00016307 | 1.000877146 | 0.004296501 |
| OIT3 | 1.139240526 | 1.086221471 | 1.19484747 | 8.26E-08 | 1.17341028 | 1.000706056 | 1.375920208 | 0.048992958 |
| TRIP13 | 1.397981227 | 1.199539333 | 1.62925171 | 1.79E-05 | 2.251435253 | 1.303299104 | 3.8893303 | 0.003617741 |
| CENPL | 1.199915285 | 1.128971876 | 1.275316704 | 4.59E-09 | 1.323373355 | 1.141284748 | 1.534513659 | 0.000207473 |
| EZH2 | 1.042096366 | 1.026147847 | 1.05829276 | 1.60E-07 | 1.062110872 | 1.022595201 | 1.103153529 | 0.001839427 |
| PTTG1 | 1.043767085 | 1.017585345 | 1.070622462 | 0.000950031 | 0.878284777 | 0.817993862 | 0.943019484 | 0.000347736 |
| DEPDC1B | 1.043433749 | 1.02837235 | 1.058715736 | 9.96E-09 | 0.952228548 | 0.908108075 | 0.998492617 | 0.043146848 |
| TRIM71 | 1.143085347 | 1.081249414 | 1.208457635 | 2.44E-06 | 0.839603204 | 0.688947273 | 1.023203906 | 0.083161967 |
| COL15A1 | 1.024544489 | 1.015951641 | 1.033210015 | 1.67E-08 | 0.961418943 | 0.931189242 | 0.992630008 | 0.01578797 |
| HAMP | 1.026001925 | 1.007123806 | 1.045233906 | 0.006745891 | 0.919701221 | 0.850734163 | 0.994259279 | 0.035315222 |
| CDK1 | 1.052036319 | 1.031347943 | 1.073139693 | 5.56E-07 | 1.120099057 | 1.056301395 | 1.187749919 | 0.000150292 |
| ZWINT | 1.079516709 | 1.041832037 | 1.118564493 | 2.44E-05 | 1.071854432 | 1.011754598 | 1.135524291 | 0.018428763 |
| NUF2 | 1.351001366 | 1.124162909 | 1.62361227 | 0.001336783 | 0.496240387 | 0.279450716 | 0.881209127 | 0.016775324 |
| FCN2 | 1.24196823 | 1.112547262 | 1.386444545 | 0.00011362 | 0.814038129 | 0.628811438 | 1.05382637 | 0.118298708 |
| CAP2 | 1.171359329 | 1.103784076 | 1.243071636 | 1.82E-07 | 1.190087282 | 0.982065422 | 1.442172496 | 0.0758413 |
| BBOX1 | 1.145527755 | 1.089443189 | 1.204499556 | 1.13E-07 | 1.725238491 | 1.405518145 | 2.117687248 | 1.84E-07 |
| TOP2A | 1.067702922 | 1.024420566 | 1.112813983 | 0.001917764 | 0.859275566 | 0.724789961 | 1.018715129 | 0.080733011 |
| PRC1 | 1.046380475 | 1.025237079 | 1.067959909 | 1.34E-05 | 0.930974361 | 0.876767055 | 0.988533108 | 0.019451316 |
| IDO2 | 1.319640977 | 1.199264907 | 1.452099781 | 1.32E-08 | 1.304586624 | 0.977862416 | 1.740476197 | 0.070643921 |
